# The Beat

**Published:** 2009-01

**Authors:** Erin E. Dooley

## In the Forecast: Better Meningitis Control in Africa

**Figure:**
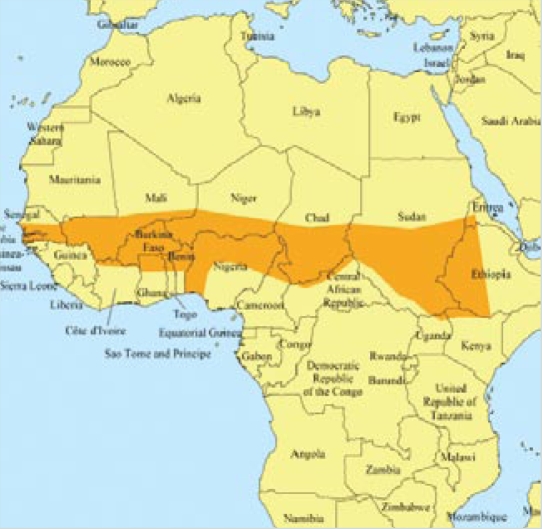
African Meningitis Belt

In Africa’s Meningitis Belt, which stretches from Senegal to Ethiopia, epidemics of the often-fatal disease occur periodically, generally beginning with the start of the dry season in December and ending with the onset of the rainy season in May. A pilot project involving an international team of scientists, funded by a grant from Google’s Predict and Prevent Program, will provide longer-term weather forecasts to health agencies in the region so they can better coordinate meningitis vaccination measures. A new vaccine being used by the agencies is currently in limited production, and vaccination efforts are hampered by the need to reach people in remote areas; this effort will help identify the populations most at risk. Though the forecasting methods being used are not new, the meteorologists involved have greater confidence in the accuracy of their predictions than in the past. The project will begin in 2009 in Ghana, a country that has been hit hard by meningitis in the past, and will then roll out to other nations.

## Dioxin Found in Irish Meat

In December 2008, Ireland recalled all pork produced in the preceding 3 months after several pork products were found to contain up to 200 times the legal limit of dioxin, a known human carcinogen. The chemical was also found in Irish beef cattle at up to 400 times the legal limit, but no beef recall was required. The dioxin was traced to a single feed supplier whose product contained dioxin-contaminated oil. In a statement issued December 10, the European Food Safety Authority declared even the most highly exposed consumers were unlikely to experience adverse health effects, given consumption levels and the uncertainty factor built into the European tolerable intake threshold for dioxin. Pork products were back on shelves by December 17.

## PM May Feed Tornado Formation

Springtime heralds the onset of tornado season across the U.S. Midwest. In Volume 35, Issue 23 (2008) of *Geophysical Research Letters*, researchers present computer models illustrating how particulate matter can increase the likelihood of tornado formation. In one model representing a relatively clean atmosphere, a rotating cloud formed, but no twister developed. In another model representing 10 times more atmospheric dust, twisters developed. The lack of particles in the “clean” model allowed rain to wash out the storm’s core, killing the downdrafts that lead to tornadoes. Coauthor David Lerach of Colorado State University says further research in real-world settings, especially in polluted urban areas, is needed to prove the hypothesis.

**Figure:**
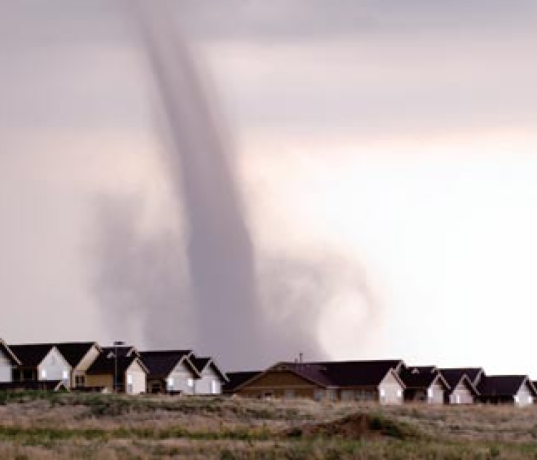


## EPA Forges Medical Waste Regs

In December 2008 the EPA announced revised regulations that will cut the amount of pollution released by U.S. medical waste incinerators States by 468,000 pounds each year. Annual emissions of mercury, a neurotoxicant, will be reduced by 637 pounds, and emissions of dioxin, a known human carcinogen, will be reduced by 40 grams. For almost every pollutant, emissions allowed from medical incinerators will be lower by at least a factor of 10.

## Seeing into the Future of CO_2_

A group of climate scientists estimates that of CO_2_ emitted from fossil fuel burning may linger in the atmosphere far longer than originally believed. Using computer models, the group calculates that more than one-third of anthropogenic CO_2_ could remain in the atmosphere for more than 2 millennia. They also confirmed earlier findings that, even if fossil fuel consumption ends, temperatures will plateau at a new higher temperature for at least 500 years. Their results appear in the 2009 *Annual Review of Earth and Planetary Sciences*.

## Phthalate Levels Decreasing in Beauty Products

*A Little Prettier*, a new report by the Campaign for Safe Cosmetics, reveals that 9 of 12 products tested had reduced or eliminated their phthalate content and that none of the products contained more than 1 phthalate. The report focused on the 12 products identified in a 2002 study as having the highest levels of phthalates. Phthalates are often used to make fragrances last longer; they are not required to be listed on product labels. Diethyl phthalate (DEP), the phthalate most commonly used in cosmetics, has been linked to developmental and reproductive effects in animal studies, and research is beginning to point toward subtle effects in humans.

**Figure:**
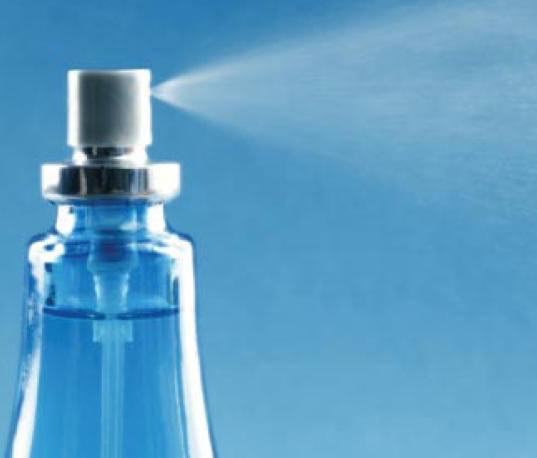
Some fragrances are using fewer phthalates.

